# A94 PERI-PROCEDURAL MANAGEMENT OF PATIENTS RECEIVING A DIRECT ORAL ANTICOAGULANT UNDERGOING A DIGESTIVE ENDOSCOPY

**DOI:** 10.1093/jcag/gwab049.093

**Published:** 2022-02-21

**Authors:** A N Barkun, C Barkun, M Martel, P INVESTIGATORS

**Affiliations:** 1 ^1.^ Gastroenterology, McGill University, The Montreal General Hospital, GI Division, Montreal, QC, Canada; 2 ^2.^ Multiple institutions, Montreal, QC, Canada

## Abstract

**Background:**

The peri-procedural management of patients on a direct oral anticoagulant (DOAC) requiring an elective digestive (GI) endoscopic procedure remains uncertain.

**Aims:**

To investigate the safety of a standardized peri-procedural DOAC management strategy.

**Methods:**

The Perioperative Anticoagulation Use for Surgery Evaluation (PAUSE) cohort study was conducted at 23 clinical centers in North America and Europe. Participants (n=3007) all had atrial fibrillation (AF), were *>*18 years old, long-term users of Apixaban, Rivaroxaban, or Dabigatran, and scheduled for an elective procedure or surgery; all could adhere to the DOAC interruption protocol. This analysis focuses on the 579 patients undergoing a digestive endoscopic procedure. The DOAC interruption (1–2 days pre-endoscopy) and resumption (1–3 days post-endoscopy) strategy is based on the DOAC molecule, patient renal function, with most GI procedures considered at low-risk for bleeding. Follow-up occurred at 30 days. Outcomes included GI bleeding and thromboembolic events (ischemic stroke, transient ischemic attack, myocardial infarction, systemic embolism, deep vein thrombosis, and pulmonary embolism) and mortality.

**Results:**

Of the 556 patients (72.5 *+*8.6 yrs; 37.4 % female), 38.9%) were on Apixaban, 36.9% on Rivaroxaban, and 24.3% on Dabigatran; 10.1% were on anti-platelet therapy. The overall CHADS score was 1.7 *+*1.0. Overall, 525 patients were categorized as having a low risk for bleeding, and 31 were at high-risk. DOAC were stopped 2.0 *+*0.5 days pre-procedure and restarted 1.9 *+*1.5 days post-procedure. Overall rates were: all bleeding 4.4% (2.9–6.4), GI bleeding 2.5% (1.4–4.2%), while 0.7% (0.3–1.8%) experienced a thromboembolic event. Additional results are listed in Table 1.

**Conclusions:**

Patients with AF undergoing a standardized DOAC therapy interruption management protocol for elective digestive endoscopy experienced low rates of major bleeding and arterial thromboembolism.

All results reported as % and 95% CI * outcomes were missing for 4 patients that had the procedure

**Funding Agencies:**

None

**Table:** 

Variable;	Patient cohort N=556	High-risk group for bleeding N=31	Low risk group for bleeding N=525*	Diagnostic procedure N=430	Therapeutic procedure N=21
Mortality	0.5 (0.2; 1.6)	0.0 (0.0; 11.0)	0.6 (0.2; 1.7)	0.5 (0.1; 1.7)	0.0 (0.0; 15.5)
Thromboembolic event	0.7 (0.3; 1.8)	0.0 (0.0; 11.0)	0.8 (0.3; 2.0)	0.9 (0.4; 2.4)	0.0 (0.0; 15.5)
All Bleeding event	4.4 (2.9; 6.4)	16.1 (7.1; 32.6)	3.7 (2.4; 5.6)	3.7 (2.3; 6.0)	19.1 (7.7; 40.0)
GI Bleed	2.5 (1.4; 4.2)	9.7 (3.3; 24.9)	2.1 (1.2; 3.7)	2.1 (1.1; 3.9)	4.8 (0.8; 22.7)

